# A framework for quantification and physical modeling of cell mixing applied to oscillator synchronization in vertebrate somitogenesis

**DOI:** 10.1242/bio.025148

**Published:** 2017-06-29

**Authors:** Koichiro Uriu, Rajasekaran Bhavna, Andrew C. Oates, Luis G. Morelli

**Affiliations:** 1 Graduate School of Natural Science and Technology, Kanazawa University, Kanazawa, 920-1192, Japan; 2 Theoretical Biology Laboratory, RIKEN, Wako, 351-0198, Japan; 3 Max Planck Institute of Molecular Cell Biology and Genetics, Dresden, 01307, Germany; 4 Max Planck Institute for the Physics of Complex Systems, Dresden, D01187, Germany; 5 The Francis Crick Institute, 1 Midland Road, London, NW1 1AT, United Kingdom; 6 Department of Cell and Developmental Biology, University College London, Gower Street, London, WC1E 6BT, United Kingdom; 7 Instituto de Investigación en Biomedicina de Buenos Aires (IBioBA) - CONICET - Partner Institute of the Max Planck Society, Buenos Aires, C1425FQD, Argentina; 8 Department of Systemic Cell Biology, Max Planck Institute for Molecular Physiology, Dortmund, 44227, Germany; 9 Departamento de Fıśica, FCEyN, UBA, Buenos Aires, 1428, Argentina

**Keywords:** Coupled oscillators, Zebrafish, Somitogenesis, Cell mixing, Imaging synchronization

## Abstract

In development and disease, cells move as they exchange signals. One example is found in vertebrate development, during which the timing of segment formation is set by a ‘segmentation clock’, in which oscillating gene expression is synchronized across a population of cells by Delta-Notch signaling. Delta-Notch signaling requires local cell-cell contact, but in the zebrafish embryonic tailbud, oscillating cells move rapidly, exchanging neighbors. Previous theoretical studies proposed that this relative movement or cell mixing might alter signaling and thereby enhance synchronization. However, it remains unclear whether the mixing timescale in the tissue is in the right range for this effect, because a framework to reliably measure the mixing timescale and compare it with signaling timescale is lacking. Here, we develop such a framework using a quantitative description of cell mixing without the need for an external reference frame and constructing a physical model of cell movement based on the data. Numerical simulations show that mixing with experimentally observed statistics enhances synchronization of coupled phase oscillators, suggesting that mixing in the tailbud is fast enough to affect the coherence of rhythmic gene expression. Our approach will find general application in analyzing the relative movements of communicating cells during development and disease.

## Abstract (precis)

**Summary:** We develop a framework to quantify and model cell mixing independent of a choice of reference frames, and apply this to study oscillator synchronization in the zebrafish segmentation clock.

## INTRODUCTION

Tissue organization in animal embryos involves relative cell movement. The importance of cell movement in development has been emphasized, for example in gastrulation, tissue elongation and neural development (Friedl and Gilmour, 2009; Rørth, 2009; Tada and Heisenberg, 2012). While on the move, cells communicate via mechanical and biochemical signalling, which can be local, for example when mediated by membrane-anchored proteins. Many developmental processes involve cell movement and local intercellular signaling simultaneously, which means that the relative durations, or timescales, of these processes may play a role in successful communication. Cells modify their internal states due to received signals and the time taken for this determines a signaling timescale. Movement that causes relative positional changes between cells is referred to as relative cell movement or cell mixing, and the time taken to exchange neighbors sets a mixing timescale. When the mixing timescale is similar to, or faster than, the local signaling timescale, cells can exchange neighbors and start new local interactions before completing the internal state change due to previous signaling events, and thus movement can affect the flow of information across a tissue (Uriu et al., 2014). However, little attention has been paid to the relation between the timescales of these two processes, or how cell mixing affects local intercellular interactions and the resulting tissue organization.

In this paper, we develop a framework to analyze and model cell mixing quantitatively using zebrafish somitogenesis as a model system, and apply the framework to determine the impact of cell mixing on synchronization of genetic oscillators. In somitogenesis, multicellular tissue blocks termed somites bud off rhythmically from the anterior end of the unsegmented tissue, which consists of the presomitic mesoderm (PSM) and, more posteriorly, the tailbud. The timing of somite formation is controlled by genes showing oscillatory waves of expression in the PSM and tailbud (Soroldoni et al., 2014). In zebrafish, these genes include *her1*, *her7* and *deltaC* (Krol et al., 2011). Oscillatory expression is thought to be caused by delayed negative feedback regulation of *her1* and *her7* (Lewis, 2003; Schröter et al., 2012). These cells have been considered and modeled as a population of noisy autonomous oscillators (Webb et al., 2016) that can interact with neighboring cells through Delta-Notch signaling (Horikawa et al., 2006; Jiang et al., 2000; Riedel-Kruse et al., 2007). Blocking Notch signaling, either using mutants or a drug that blocks the activation of the Notch receptor (DAPT), revealed that synchronized oscillation of gene expression is necessary to make normal somites (Delaune et al., 2012; Liao et al., 2016; Mara et al., 2007; Özbudak and Lewis, 2008; Riedel-Kruse et al., 2007). Delta-Notch signaling also maintains synchronization between PSM cells in mouse embryos (Okubo et al., 2012; Shimojo et al., 2016) and tissue cultures (Tsiairis and Aulehla, 2016). The collective rhythm arising from Delta-Notch interaction across the PSM is the temporal signal of a ‘segmentation clock’ (Liao et al., 2016; Oates et al., 2012; Pourquié, 2011; Shimojo and Kageyama, 2016). In posterior PSM and tailbud, oscillation phase is spatially uniform, synchronized across the cell population.

Cells carrying the genetic oscillators move around, exchanging neighbors in posterior PSM and tailbud (Bénazéraf et al., 2010; Delfini et al., 2005; Dray et al., 2013; Kulesa and Fraser, 2002; Lawton et al., 2013; Mara et al., 2007). Previous experiments focused on the role of cell movement in axis elongation using time-lapse imaging in zebrafish (Dray et al., 2013; Lawton et al., 2013; Mara et al., 2007; Steventon et al., 2016) and chick (Bénazéraf et al., 2010; Delfini et al., 2005). Cells in PSM and tailbud extend protrusions (Bénazéraf et al., 2010; Manning and Kimelman, 2015), and are thought to possess intrinsic motility. These studies also revealed signaling molecules driving cell movement in posterior PSM and tailbud of chick. Fgf forms a spatial gradient across the PSM with highest concentration in the tailbud (Dubrulle and Pourquié, 2004), and activates cell movement (Bénazéraf et al., 2010; Delfini et al., 2005). Cells in anterior PSM show reduced cell movement due to low levels of Fgf signaling and epithelialization (Delfini et al., 2005). Combined, these experimental observations raise the question of how cell mixing in posterior PSM and tailbud influences synchronization of genetic oscillators.

Previous theoretical studies suggested that cell mixing in the tailbud could promote synchronization across a population of genetic oscillators (Uriu et al., 2012, 2010; Uriu and Morelli, 2014). Movement of oscillators can effectively extend their interaction range (Fujiwara et al., 2011; Peruani et al., 2010; Uriu, 2016; Uriu et al., 2013). However, an enhancement of synchronization is only possible if the timescale of cell mixing is faster than the timescale of cell signaling. These previous theoretical studies assumed such faster cell mixing and analyzed its effect on synchronization of oscillators. While the timescale of cell signaling has been estimated from experiments in which synchronization is perturbed by blocking Notch with DAPT (Herrgen et al., 2010; Riedel-Kruse et al., 2007), the timescale of cell mixing has not been measured. Previous studies of cell movement provided measurements of velocity and mean squared displacement (MSD) of single cells (Bénazéraf et al., 2010; Lawton et al., 2013), but how often cells exchange neighbors has not yet been quantified. For this, knowledge of the cells' velocity is not sufficient; rather the relative motion of cells is required. Furthermore, direct comparison between mixing and signaling timescales is not trivial because complex cell movement patterns in the zebrafish tailbud (Lawton et al., 2013) may prevent characterization of cell mixing with a single timescale (Uriu and Morelli, 2017). Hence, a method to deal with these challenges rigorously and systematically needs to be developed.

Here, we propose a framework motivated by the question of whether cell mixing in the zebrafish PSM is fast enough to affect synchronization of genetic oscillators. This starts with quantifying cell mixing across zebrafish PSM and tailbud using embryonic time-lapse images at single cell resolution. To characterize cell mixing, we compute spatial derivatives of cell velocities and mean squared difference of displacement vectors (MSDD) (Uriu and Morelli, 2014) from cell-tracking data. This removes any global tissue motions in the imaging reference frame and yields the relative motion of cell pairs. Then, we fit a physical model of cell movement and reproduce the cell mixing observed across the tissue. Finally, we simulate synchronization dynamics of coupled phase oscillators in the presence of reproduced cell mixing and show that the reproduced cell mixing enhances synchronization. Thus, the proposed approach gives a general and systematic framework to quantitatively analyze cell mixing in development. Its application suggests that cell mixing in zebrafish tailbud is indeed fast enough to affect synchronization dynamics of the segmentation clock.

## RESULTS

### Single cell tracking

**Fig. 1.**
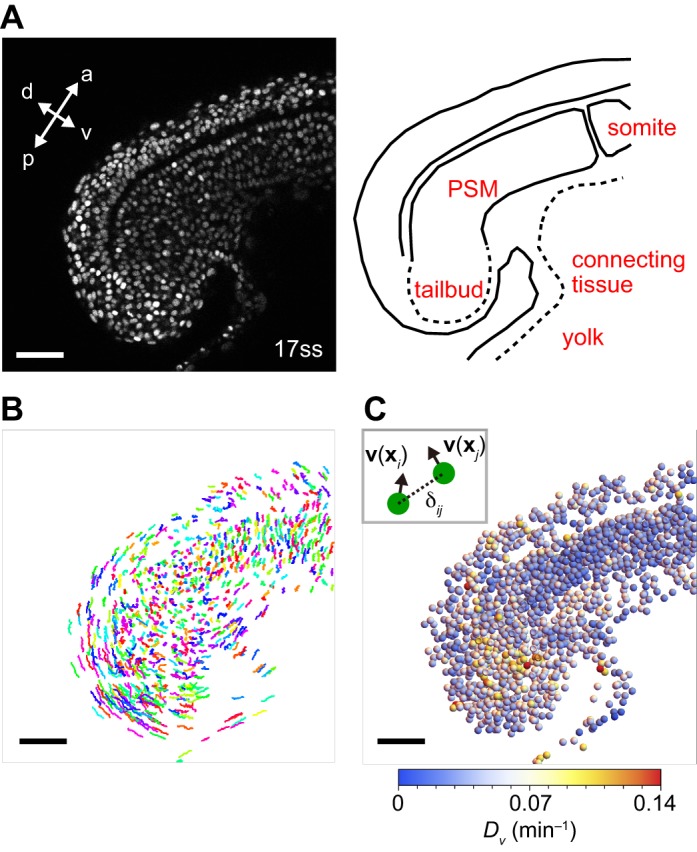
**Quantification of cell mixing by the average directional derivative modulus of cell velocity vectors.** (A) Left: Snapshot of a 17 ss embryo, with nuclei labeled with Histone *h2AflV-gfp*. a, anterior; p, posterior; d, dorsal; v, ventral. Right: schematic picture of PSM and tailbud. See also Movie 1. (B) Cell trajectories for the embryo shown in A, obtained by nuclear detection and tracking algorithms. Trajectories from time frame 1 to 20 (16.7 min) plotted in three-dimensional space. Each trajectory is assigned a color randomly. (C) Spatial profile of average directional derivative modulus of cell velocity vectors *D_v_* for the 17 ss embryo shown in A. Spheres represent the positions of cells (radius chosen for better visibility). Scale bars: 50 μm.

Cell movement can be estimated using the position of each cell's nucleus as a reference point. The nuclei of cells in tailbud, PSM and posterior somites in zebrafish embryos (*n*=4) were imaged with high temporal resolution for an interval corresponding to the formation of one somite, starting at the 15-17 somite stage (ss), from a lateral orientation by confocal microscopy using a setup for multiple-embryo time-lapse recording (Fig. 1A; Movie 1) (Bhavna et al., 2016). To detect the position of each nucleus, we used the gradient vector diffusion algorithm proposed by Li et al. (2007). For cell tracking, we adopted an algorithm based on nearest neighbor linking of objects between two successive time frames *t* and *t*+1 (Fig. 1B) (Sbalzarini and Koumoutsakos, 2005).

### Validation of cell tracks

Embryos in this study were transgenic chimeras in which cells carrying both mCherry and GFP-tagged Histones as nuclear labels were transplanted at blastula stage to stage-matched host embryos carrying only GFP-Histone. The sparsely distributed mCherry nuclear signal was an internal ground-truth data set (Bhavna et al., 2016) to allow validation of our nuclear detection and cell-tracking algorithms (Supporting Text) (Bhavna et al., 2016). Parameters in the gradient vector diffusion algorithm were determined by calibration using synthetic images with similar nuclear density and image signal-to-noise ratios to our embryonic data. To quantify accuracy, we defined sensitivity as the fraction of objects correctly detected by the algorithm to the total number of objects in a synthetic image, and precision as the fraction of correctly detected objects to the total number of detected objects (Supporting Text). The sensitivity of the algorithm with optimized parameter set was ∼90% and precision was ∼95% in synthetic images with relevant object densities (Fig. S1A). Sensitivity of the algorithm in transplanted embryos ranged between 0.96 and 0.98 (Fig. S1B). The fraction of cells with incorrect trajectories was low (0-2%) (Fig. S1C,D). Although the tracking algorithm occasionally missed cells at some time point, resulting in a trajectory shorter than the recording's length (Fig. S1E), this does not lead to incorrect calculations of cell displacements in later analysis, which arise primarily from incorrect linking.

### Cell mixing

A key property of cell movement that affects synchronization is local rearrangement, which will result in the mixing of neighboring oscillators (Uriu and Morelli, 2014). From cell trajectories it is straightforward to compute cell velocity. However, velocity computed in the laboratory reference frame includes contributions of spontaneous cell movement and also global tissue motion: embryos can move on the microscope stage, and the body axis deforms and elongates as a result of normal development. Consequently, velocity vectors in the laboratory reference frame do not reveal relative positional changes of cells. Below, we introduce two different methods to quantify cell mixing, namely the directional derivative of velocity vectors and the MSDD.

#### Directional derivative of velocity vectors

Local cell rearrangement may be quantified by the velocity difference of neighboring cells. A large velocity difference indicates that neighboring cells move in different directions resulting in relative positional changes. We compute the difference of velocity vectors for a pair of neighboring cells *i* and *j* at position **x***_i_* and **x***_j_* as(1)
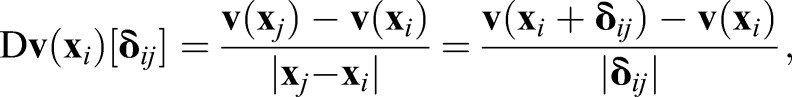
where **δ***_ij_*=**x***_j_*−**x***_i_*. Eqn 1 approximates the spatial derivative of velocity vectors along vector **δ***_ij_*. We refer to D**v**(**x***_i_*)[**δ***_ij_*] as the directional derivative. To determine the magnitude of local velocity variations at cell position **x***_i_*, we compute the average of directional derivative modulus over neighboring cells:(2)
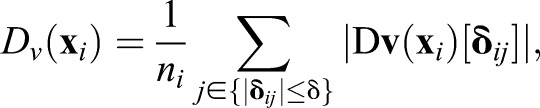
where *n_i_* is the total number of neighboring cells satisfying |***δ****_ij_*|≤*δ* and summation is over all neighboring cells *j*. By subtracting two neighboring cells' velocities, the components of velocity drifts due to embryonic movement and tissue deformations are cancelled out, and only components due to relative movement remain. Thus, *D_v_* is a proxy for the magnitude of cell mixing.

Fig. 1C shows the spatial profile of *D_v_* along the PSM of a 17ss embryo. Based on the cell diameter estimated from the embryonic images (Fig. S2A, Supporting Text), we set *δ* =16 μm in Eqn 2. The spatial gradient of *D_v_* is highest at the posterior and progressively decreases in the anterior direction. Greater local velocity variations are observed in most cells in the tailbud, indicative of cell mixing, whereas few cells in anterior PSM have high values of *D_v_*. These higher values may be local fluctuations of velocity vectors due to cell intercalations or extrusions. In addition, relatively higher *D_v_* can be observed in cells in the connecting tissue between embryo and yolk because of this tissue's local deformation. We observed a similar spatial profile of *D_v_* over time in the embryo (Fig. S3A-C). Spatial profiles of *D_v_* among different embryos were quantitatively similar (Fig. S3D-F). Thus, the average directional derivative modulus indicates the presence of high cell mixing in the tailbud. We also quantified local velocity variations using strain rate tensor along the axis (Supporting Text) and obtained qualitatively similar spatial profiles of the magnitude of mixing (Fig. S4).

#### MSDD

The directional derivatives contain information about short timescales of cell movement. To explore long-time signatures of the movement pattern and reveal whether the cells' motion is relevant for synchronization, we introduced MSDD (Gerlich and Ellenberg, 2003; Uriu and Morelli, 2014). Using nuclear positions **x***_i_* obtained by the tracking algorithm, MSDD *m*(*t*) was defined as:(3)
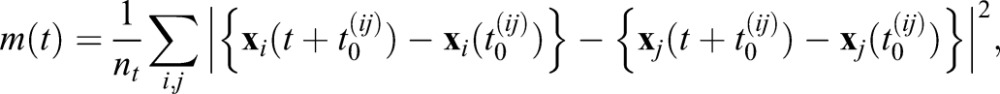
where *t*_0_^(*ij*)^ is the time when cells *i* and *j*, for the first time, satisfy |**x***_i_*(*t*_0_^(*ij*)^)–**x***_j_*(*t*_0_^(*ij*)^)|≤*r* in the imaging period and *n_t_* is the total number of pairs with the value *t*. Note, the value of *t*_0_^(*ij*)^ can be different for each pair of cells *i* and *j*. We set the distance threshold for averaging *r*=16 μm, which is close to measured cell size (Fig. S2A). This restricts cell pairs to initial neighbors, avoiding the contribution of spatially heterogeneous tissue motions. The relation between MSDD and MSD is described in Uriu and Morelli (2017).

**Fig. 2.**
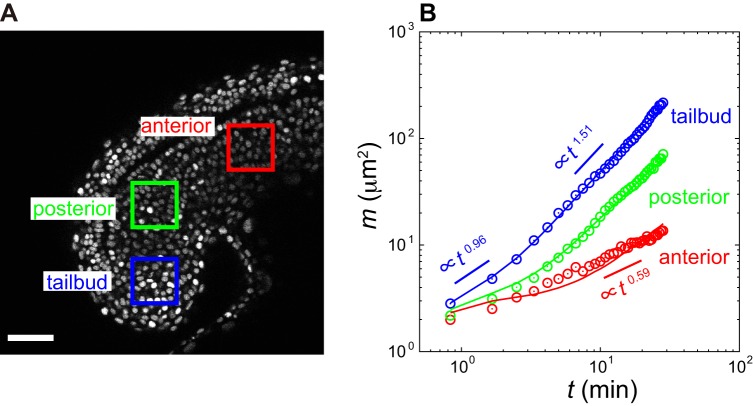
**Quantification of cell mixing by MSDD.** (A) Snapshot of a 17 ss embryo. Colored boxes indicate regions for which MSDD was calculated in B. (B) MSDD computed from Eqn 3 at each region of PSM and tailbud as a function of time. Circles represent experimental data. Lines are fit by the physical model of cell movement to experimental data. Tables S1 and S2 give parameter values in the physical model. Scale bar: 50 μm.

Fig. 2 shows time evolution of MSDD in three selected regions of a 17ss embryo. We set a three-dimensional box in a local region (Fig. 2A) and used cells within the box to compute MSDD defined in Eqn 3 (Materials and Methods). MSDD increased more rapidly in the posterior region than in the anterior, which indicated that relative cell movement was faster in the posterior region than in the anterior region (Fig. 2B). This is consistent with analysis of directional derivative of velocity vectors (Fig. 1; Fig. S3) and strain rate tensor (Fig. S4) described above. We observed two regimes in MSDD curves. If cell movement was a random walk, we expect a linear increase of MSDD over time (Uriu and Morelli, 2017). For cells in the tailbud, MSDD increased almost linearly *m*(*t*)∝*t* at shorter time (*t*<3 min) while at longer time (*t*>3 min) it increased as a power law of *t*, *m*(*t*)∝*t*^1.5^. This exponent indicates that cell movement in zebrafish tailbud is not a simple random walk, in contrast to reported movements in chick embryos (Bénazéraf et al., 2010). Note that this two-phase behavior of MSDD cannot be explained by a persistent random walk model because its MSDD should behave as *m*(*t*)∝*t*^2^ at shorter time (Gardiner, 2009). To confirm this behavior, we applied a second, recently proposed segmentation algorithm (Bhavna et al., 2016) and obtained similar results (Fig. S5).

In regions more anterior to the tailbud, we observed a similar tendency of the MSDD, but values of exponents decreased to less than one, indicating sub-diffusive cellular motions (Fig. 2B). We obtained quantitatively consistent MSDD among the other three embryos at similar developmental stages (Fig. S6).

Power law behaviors of MSDD described above preclude computation of a single timescale of cell mixing such as the diffusion constant of cells. This makes it difficult to directly compare the timescale of cell mixing and that of the phase dynamics of genetic oscillators (Uriu et al., 2013). To overcome this difficulty, we developed a physical model of cell movement to reproduce the observed mixing in zebrafish embryos. Since cell tracking was performed using nuclear positions, we hypothesized that linear increase of MSDD at shorter time reflects motion of nucleus within cytoplasm, while power law increase at longer time indicates persistent cell movement constrained by neighboring cells. We tested this hypothesis by fitting the physical model to the MSDD data obtained from embryonic images.

### Modeling cell movement

**Fig. 3.**
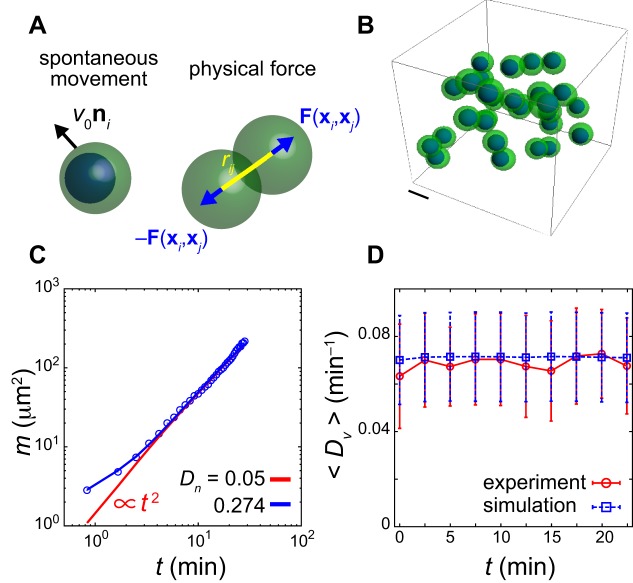
**Physical model for cell movement.** (A) Left: cell in a three-dimensional space is represented as a sphere (green). Dark-blue sphere inside indicates cell nucleus. The unit vector **n***_i_* represents polarity for spontaneous cell movement. Right: repulsive physical forces between two neighboring cells. (B) Snapshot of a simulation: 30/346 cells are plotted. Scale bar: 10 μm. See Movie 2. (C) MSDD as a function of time. Lines indicate simulation results for different values of nuclear diffusion constant *D_n_*. Circles are experimental data for tailbud cells in Fig. 2B. (D) Time series of population average of directional derivative modulus *D_v_* for embryonic tailbud region (red circles; experiment) and for simulations of the fitted physical model (blue squares; simulation). Embryonic data in Fig. 1C and Fig. S3 were used to compute population average of *D_v_*. Error bars indicate standard deviations.

We chose a description of cell movement in PSM and tailbud allowing for direct comparison between timescales of cell mixing and oscillator phase dynamics. Because nuclei can move within cytoplasm and MSDD was computed with nuclear positions, the model describes movement of both cells and nuclei. Cells were described as spheres of diameter *d_c_* in a confined three-dimensional space representing a local region somewhere in PSM or tailbud (Fig. 3A). The number of cells *N* in the model was set to fit cell density observed in embryos (Fig. S7, Supporting Text). We did not consider cell proliferation and apoptosis in the model. A similar description of a cell population was previously used to study synchronization dynamics (Tiedemann et al., 2012, 2007, 2014). However, this previous model did not consider cell movement.

We assumed that cells are self-propelled particles experiencing physical contact forces between them. We wrote the over-damped equation of motion for the cell center **x***_i_*(*t*) (*i*=1, 2, …, *N*) (Uriu and Morelli, 2014):(4)
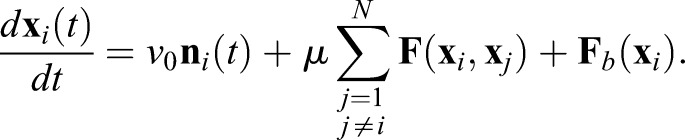
The first term describes spontaneous movement of cells. Without forces, cells move in direction **n***_i_* at speed *v*_0_. This direction of spontaneous motion **n***_i_* is a vector performing random walk on a unit sphere. Note that a cell moving at the instantaneous velocity *d***x***_i_*/*dt*=*v*_0_**n***_i_* possesses a finite persistence of direction of motions, as reported previously (Lawton et al., 2013; Manning and Kimelman, 2015). The second term describes volume exclusion forces between neighboring cells with a strength given by *µ*. Two cells at a distance closer than cell diameter *d_c_* repel each other (Fig. 3A). The third term is the confinement force exerted by the domain boundaries.

Since we tracked cell nuclei in embryonic imaging data, we explicitly model nuclear motion inside a cell to consider its contribution to MSDD (Fig. 3A). Each nucleus is represented as a sphere of radius *r_n_*. We assumed that movement of the cell nucleus was random with a diffusion constant *D_n_*, and confined to the cytoplasmic region within the cell diameter. See Supporting Text for implementation of the model.

Fig. 3B and Movie 2 show a simulation of the physical model. Note, we plotted only a subset of total cells in the simulation in Fig. 3B and Movie 2 for better visibility. The simulation had the same cell density as the actual tailbud (Fig. S7). We found that nuclear diffusive motions in the cytoplasm explained the linear increase of MSDD at shorter time (Fig. 3C). The nucleus did not move when the nuclear diffusion constant *D_n_* was small. In such cases, instead of *m*(*t*)∝*t*, MSDD at shorter time increased as *m*(*t*)∝*t*^2^ capturing short-time persistence of cell body motions (Fig. 3C). Thus, our physical model suggests that linear increase of MSDD at early times is caused by nuclear motion within a cell. In anterior PSM, the exponent of MSDD was <1 (Fig. 2B). This observation implies that both cell and nuclear movement become slower as cells leave the posterior PSM. In simulations in Fig. 3C, the power law increase in MSDD at longer time is due to the presence of a crossover between directed cellular motions at a shorter timescale and random motions at a longer timescale.

### Fitting the physical model to embryonic MSDD data

To fit this physical model to experimentally obtained MSDD data in Fig. 2B, we adopted Approximated Bayesian Computation based on Markov chain Monte Carlo (ABC MCMC; Supporting Text) (Csilléry et al., 2010; Sunnåker et al., 2013). ABC has previously been used to fit mathematical models to experimental data (Cohen et al., 2014; Marjoram et al., 2003). We computed MSDD in simulations using nuclear position for each cell. We defined the distance *d_s_* between MSDD in simulation and experiment (Supporting Text). If *d_s_* is small for a given parameter set, the simulation explains the experimental data well. ABC MCMC allows parameters in the model to be sampled from a conditional probability distribution *P*(*ϑ*|*d_s_*≤*ε*), where *ε* represents a tolerance for fitting and *ϑ* represents the parameter set in the physical model.

We obtained values of cell density *ρ*, cell diameter *d_c_* and nuclear radius *r_n_* by direct measurement from embryonic images (Figs S2 and S7; Supporting Text). The model includes six additional free parameters determined by ABC MCMC (Fig. S8A,B). We first focused on the tailbud. For illustration, we show that choosing a parameter set yielding a small value of *d_s_* allowed the model to capture the features of the MSDD curve obtained by cell tracking in embryos (Fig. 2B; Fig. S6, Tables S1 and S2). Moreover, the fitted model could also reproduce the population average of directional derivative modulus observed in the tailbud (Fig. 3D), which was not used in ABC MCMC fitting. Using the fitted model we estimated single-cell speed and velocity auto-correlation in the tailbud (Fig. S9).

To check the model's consistency, we asked if the same model could reproduce the MSDD curves observed in anterior PSM. Given that the magnitude of cell mixing forms a spatial gradient across the PSM (Figs 1 and 2) (Bénazéraf et al., 2010; Delfini et al., 2005), we tuned the value of *v*_0_ while matching the observed cellular density and fitted MSDD in anterior regions with all other parameters fixed at their values from the tailbud (Fig. 2B). The fitting became more difficult in anterior than in posterior regions, perhaps because the diffusion constant of the nucleus may also change along the PSM as cells become nonmobile in anterior regions. However, overall, the physical model could reproduce the MSDD observed in experiments in different regions of the PSM well, with changes only to *v*_0_ and the measured density (Fig. 2B). We also confirmed that the physical model with similar parameter values could reproduce MSDD in the other three imaged embryos (Fig. S6).

### Synchronization of coupled mobile phase oscillators

Applying the physical model, we investigated whether the observed tailbud cell mixing would be fast enough to affect segmentation clock synchronization. We simulated a coupled phase oscillator model to follow the dynamics of synchronization. Each oscillator resides on a cell in the physical model Eqn 4, which allows us to reproduce the experimentally observed cell mixing (reproduced mixing). Following previous studies (Kuramoto, 1984; Morelli et al., 2009; Riedel-Kruse et al., 2007; Uriu and Morelli, 2014), we introduced a population of phase oscillators *θ_i_* (*i*=1, 2,…, *N*) with autonomous frequency *ω_i_*. The autonomous frequency obeys a normal distribution *ω_i_*∼*N*(*ω*_0_, *σ_ω_*), where *ω*_0_ is mean and *σ_ω_* is standard deviation of the distribution. We approximated the value of *ω*_0_ from the somitogenesis period at our imaging temperature (40 min at 23°C) (Schröter et al., 2008). We assumed that cells signal to those cells touching them, i.e. when the distance between them is less than the cell diameter |**x***_j_*(*t*)−**x***_i_*(*t*)|≤*d_c_*. The equation for phase oscillators reads(5)
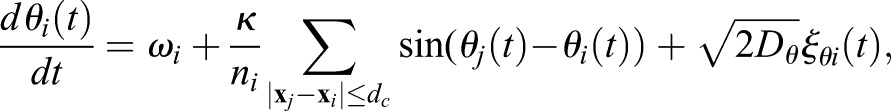
where *κ* is coupling strength between oscillators, *n_i_* is the number of contacting cells for cell *i*, *D_θ_* is phase noise strength and *ξ_θi_* is white Gaussian noise with 〈*ξ_θi_*(*t*)〉=0 and 〈*ξ_θi_*(*t*)*ξ_θj_*(*t*′)〉=*δ_ij_δ*(*t*−*t*′). We adopted an open boundary condition in simulations of phase oscillators.

The key parameter in the coupled phase oscillator model is coupling strength *κ*, setting the timescale of change in phase due to interactions, 1/*κ*. If the timescale of cell mixing is slower than 1/*κ*, synchronization dynamics is almost the same as for nonmobile cells (Uriu et al., 2013). To examine how the effect of the observed mixing depends on coupling strength, we changed its value within a plausible range from *κ*=0.01 min^–1^ to 0.11 min^–1^ (Table S3), consistent with experimental estimates (Herrgen et al., 2010; Riedel-Kruse et al., 2007). Single-cell level observations of relatively slow resynchronization after cell divisions (Delaune et al., 2012) also support the above choice of the upper bound of the coupling strength.

To explore the effect of cell mixing, we compared synchronization dynamics of oscillators in the presence of reproduced mixing for the tailbud to that of nonmobile oscillators. To quantify the degree of phase synchronization in simulations, we introduced the Kuramoto phase order parameter (Kuramoto, 1984):(6)
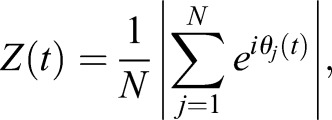
where 
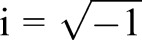
. When oscillators are synchronized, the value of the order parameter is almost 1, whereas when they are not, its value is close to 0.

During normal somitogenesis, the oscillators of the segmentation clock must maintain their phase synchronization in the presence of noise (Horikawa et al., 2006; Jiang et al., 2000; Riedel-Kruse et al., 2007). We first confirmed that the reproduced mixing could enhance robustness of the synchronized state against phase noise (Fig. S10A-C and Fig. S11).

**Fig. 4.**
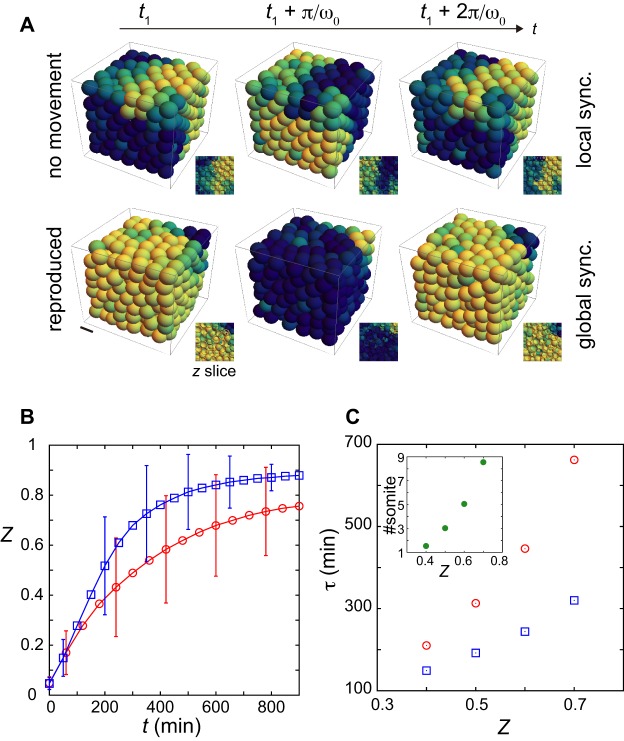
**Synchronization promoted by the reproduced cell mixing.** (A) Snapshots of spatial phase profiles emerging from random initial conditions in the phase oscillator model. The top row shows results without mixing (*v*_0_=0.14), the bottom row shows those with reproduced mixing (*v*_0_=1.39), from the tailbud in Fig. 2B. Snapshots during one oscillation cycle (2π/*ω*_0_=40 min) are shown, *t*_1_=213 min. Color code indicates phase of oscillation. Scale bar: 10 μm. See Movies 3 and 4. (B) Time evolution of phase order parameter *Z* from random initial phases in the presence of reproduced mixing as in A (blue) and in the absence of mixing (red). (C) First passage time *τ* (vertical axis) of a given value of phase order parameter (horizontal axis) for data in B. Inset: differences of first passage times Δ*τ* divided by segmentation clock period (40 min; vertical axis, #somite) as a function of the phase order parameter *Z*. Error bars in B indicate standard deviations of *Z* over 200 realizations of simulations. *κ*=0.07 min^–1^. All other parameter values in Eqns 4 and 5 are listed in Tables S1-S3.

We next asked how cell mixing affects dynamics towards the synchronized state. We simulated time evolution of *Z* from random phases at initial time, which represents the situation in which the oscillators have been desynchronized by some external perturbation, for example a DAPT ‘wash-out’ experiment (Liao et al., 2016; Riedel-Kruse et al., 2007). In the presence of DAPT, cells lose coupling and their phases desynchronize due to noise (Riedel-Kruse et al., 2007). After DAPT is washed out, Delta-Notch signaling works again and cells rebuild coherent oscillations from random phases. Fig. 4A and Movies 3 and 4 show the spatial phase profiles developed from random initial phases in simulations. For illustration we set *κ*=0.07 min^–1^, a value within the estimated range for the coupling strength (Herrgen et al., 2010; Riedel-Kruse et al., 2007). Nonmobile cells (top row Fig. 4A; Movie 3) first formed local phase synchronization, which persisted and prevented the system from attaining global synchronization. Mobile cells also first formed local synchronization, but could then relax these local phase patterns and reach global synchronization quicker (bottom row Fig. 4A; Movie 4).

The phase order parameter *Z* increased faster with reproduced mixing than without mixing (Fig. 4B), suggesting that observed cell mixing in tailbud could affect synchronization of coupled genetic oscillators *in vivo*. At short timescale (<∼100 min) the values of *Z* were almost the same between these two cases. During this period, oscillators quickly developed spatial phase patterns by local interactions. However, at around *t*=300 min, we observed a difference in *Z* between these two cases. Although different parameter sets in the model for cell movement could reproduce MSDD data in tailbud (Fig. S8), we confirmed that time evolution of *Z* was comparable for similar MSDD time series (Fig. S8F). Thus, the specific values of parameters in the physical model are not critical, but the rate of MSDD increase determines synchronization dynamics of mobile coupled oscillators. We also confirmed that cell mixing in the tailbud of the other three imaged embryos enhanced synchronization (Fig. S12). For low coupling strength (*κ*=0.03 min^–1^), the effect of mixing could be seen more clearly when simulations were started from random initial phases (Fig. S10D). Even for the largest tested coupling strength (*κ*=0.11 min^–1^), we observed improvement by the reproduced mixing (Fig. S10F). Thus, within the estimated range of the coupling strength, observed cell mixing enhanced synchronization of oscillators.

In previous experimental studies, recovery of synchronization was quantified by the time taken for a normal somite to form after DAPT wash-out (Liao et al., 2016; Riedel-Kruse et al., 2007). This recovery time represents the time taken for the phase order parameter to surpass a certain threshold value *Z_c_*: normal somites form when *Z*≥*Z_c_*. Using the simulated time series shown in Fig. 4B, we computed the first passage time *τ* of a given value of *Z* (Fig. 4C). The difference of first passage time between nonmobile and mobile oscillators became larger as *Z* increased. The time taken to reach *Z_c_* can be measured in units of the 40-min cycle of the clock, which represents the number of defective segments. The observed differences in the number of segment defects are displayed in Fig. 4C (inset). For example, for *Z_c_*∼0.7, without movement the embryo will make ∼8 more defective segments than with reproduced mixing. Hence, the physical model predicts that recovery time of correct somite boundary formation would be strongly influenced by cell mixing.

Taken together, these results suggest that there is a biologically plausible range of coupling strength in which the reproduced cell mixing significantly promotes synchronization of coupled phase oscillators. Thus, our quantification of mixing in the developing zebrafish embryo combined with theoretical modeling supports the hypothesis that cell mixing in the tailbud may promote synchronization of the segmentation clock.

## DISCUSSION

Previous studies on cell movement in development have often focused on the role of relative cell movement in perturbing patterns established by signaling systems. Examples include effects of cell divisions and intercalations on tissue boundary formation in *Drosophila* wing disc and vertebrate hindbrain (Dahmann et al., 2011). In these and similar cases, cell mixing decreases the reliability of the pattern, and mechanisms have been discovered that restrict mixing at the boundary. In contrast, local cell-sorting can correct an initial spatially noisy specification of cell types to a sharp boundary (Xiong et al., 2013). In the segmentation clock, the synchronization of noisy neighboring oscillators is a key step in the generation of a coherent pattern that leads to reliable somite boundaries at the anterior end of the PSM (Delaune et al., 2012; Jiang et al., 2000; Riedel-Kruse et al., 2007). How mixing of cells in PSM and tailbud affects this patterning system is not yet understood.

Here, we developed a framework to analyze and model cell mixing in embryonic tissues, and used a quantitative model to investigate whether the observed mixing in the zebrafish tailbud could affect synchronization of genetic oscillators. We computed directional derivatives of velocity vectors and MSDD to quantify cell mixing across PSM and tailbud (Figs 1 and 2). Then, we fitted a physical model of cell movement to experimental data and reproduced this cell mixing in simulations (Figs 2 and 3). Finally, by simulating a coupled phase oscillator model (Fig. 4) with previously estimated coupling parameter values, we showed that the reproduced mixing was fast enough to promote synchronization.

Setting a reference frame for cell movement is key to quantification, otherwise global tissue movements influence analysis. Previous studies quantified cell movement in PSM and tailbud to examine its influence on axis elongation (Bénazéraf et al., 2010; Dray et al., 2013; Lawton et al., 2013). These previous studies used extracellular matrix (Bénazéraf et al., 2010) or position of the anterior PSM (Lawton et al., 2013) to set the reference frame. The average position of tracked cells has also been used as a local reference frame when cell movements are confined within a smaller region of the tissue (Xiong et al., 2013). Alternatively, image registration algorithms (Annila et al., 2013; Qu et al., 2015) may remove cell displacements caused by embryonic motions. In this study, we take a simpler and more direct approach that focuses on relative motions, and does not rely on a choice of reference frame by adopting the spatial derivative of velocity vectors and the difference of displacement vectors, the MSDD.

We observed two different regimes of MSDD in 15-17ss embryos imaged at 23°C. At shorter times, MSDD increased almost linearly over time. We explained these shorter time behaviors by nuclear motions (Fig. 3C). Indeed, diffusive nuclear motions in the cytoplasm have been observed in mesenchymal cells migrating on a two-dimensional substrate (Liu et al., 2015). At longer times, MSDD increased as a power law with an exponent larger than one. We explained this power law increase by persistent cell movement (Fig. 3; Fig. S9). A previous study using zebrafish embryos at 10ss growing at 18°C showed that MSD for single cells in the tailbud increases as a power law of time and that the exponents are larger than one (Lawton et al., 2013). MSDDs from this data set determined with our methods also showed power law exponents greater than one (Fig. S13), and were similar to those for the 15-17ss embryos imaged in the present study. Thus, both previous and present studies indicate that cell movement is not a simple random walk in zebrafish posterior PSM. Furthermore, the similar rate of MSDD increase observed in those 10ss embryos (Fig. S13) suggests that cell mixing at this earlier developmental stage would also influence synchronization of oscillators.

Previous theoretical studies examined the effect of cell mixing on synchronization of genetic oscillators in the tailbud with an assumption that cell mixing timescale is faster than signaling timescale defined by the inverse coupling strength 1/*κ* (Uriu et al., 2012, 2010; Uriu and Morelli, 2014). This critical assumption, however, has not been tested experimentally. In general, complex cell movement patterns in developing tissues would exclude the characterization of cell mixing with a single timescale, as shown in Fig. 3 (Uriu and Morelli, 2017). The framework proposed here can predict the impact of observed cell mixing on signaling even when cell mixing and signaling includes multiple timescales. Current and previous modeling (Uriu and Morelli, 2014) indicate that a main determinant of synchronization dynamics is the rate of MSDD increase (Fig. S8). This is an increasing function of the ratio *v*_0_/*µ* in Eqn 4 and its estimated values are within the range of 0.16-0.3 (Table S1). Although these obtained values are smaller than those assumed in a previous study (Uriu and Morelli, 2014), the observed mixing does enhance synchronization in this range (Fig. 4; Fig. S12). Collective behaviors of mobile interacting agents are relevant to not only biology but also physics (Fujiwara et al., 2011; Levis et al., 2017; Peruani et al., 2010) and technology (Wang et al., 2009). Determining whether the mobility of agents is faster than the timescale of interactions is an important step to analyze such systems as well.

**Fig. 5.**
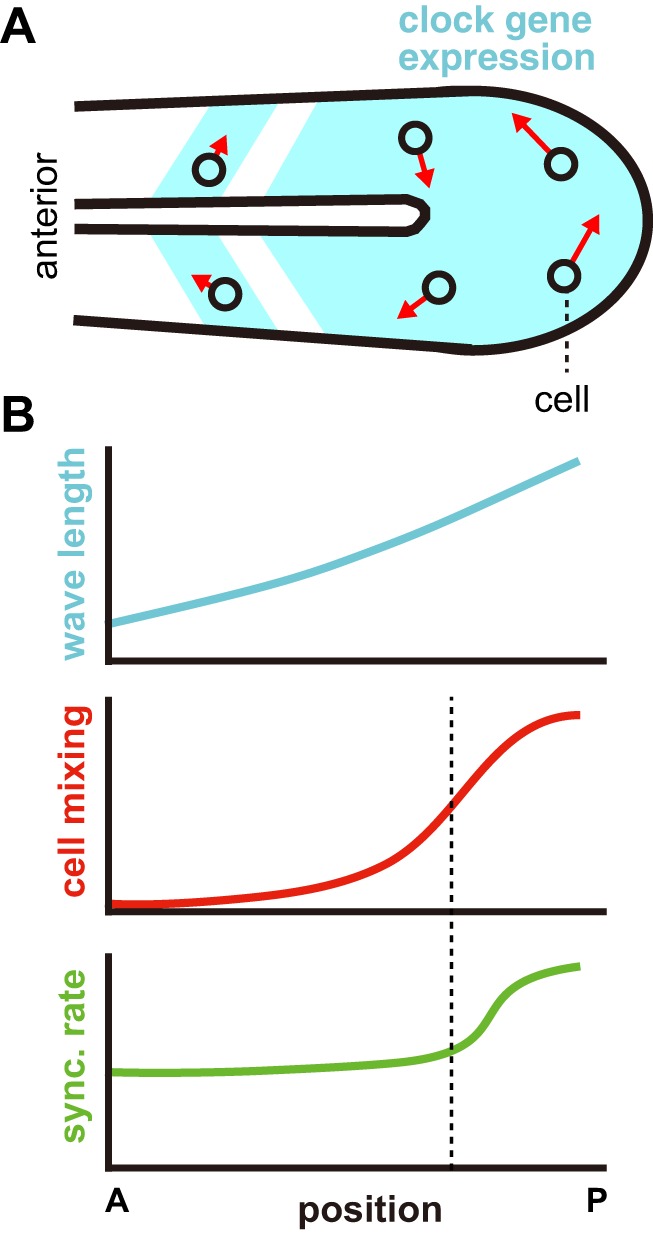
**Robust somite boundary formation by cell mixing gradient.** (A) Expression pattern of a segmentation clock gene in PSM and tailbud. The red arrows indicate cell velocity vectors and their lengths represent velocity moduli. (B) Spatial gradients of wavelength of a gene expression pattern (top), cell mixing (middle) and predicted synchronization (sync.) rate (bottom) along the anterior-posterior axis of the PSM. Vertical dashed line indicates the position where cell mixing can no longer affect the synchronization of genetic oscillators. A, anterior; P, posterior.

A striking feature of the data is the gradient of cell mixing, highest in tailbud and lowest in anterior PSM, as previously noted (Bénazéraf et al., 2010; Lawton et al., 2013). One implication of our findings is that there may exist a threshold in the PSM at which cell mixing is no longer beneficial for synchronization (Fig. 5). Oscillations in PSM are organized as waves of gene expression that sweep from posterior to anterior. A wave slows as it moves anteriorly and stops where the next somite boundary will form (Aulehla et al., 2008; Soroldoni et al., 2014). Accordingly, the wavelength of the gene expression stripes becomes shorter in the anterior PSM, approaching that of the somite length. If cells moved faster than gene expression waves, stripe boundaries would be blurred. Thus, slow cell mixing observed in the anterior is consistent with the formation of sharp somite boundaries. In contrast, the effective interaction range (Uriu et al., 2013) introduced by fast cell mixing in the tailbud is smaller than the large wavelength spanning this region (Soroldoni et al., 2014) and smaller than tailbud size (Fig. S14; Supporting Text). Robust synchronization by cell mixing in the tailbud (Figs S10 and S11) is important because cells leave the tailbud carrying their local phase order and emerge into the PSM, where a failure in synchronization causes local defects in the gene expression stripes, resulting in defective segment boundary formation.

A second implication is that the mixing of cells may itself influence the wave pattern. Synchronized cells leave the tailbud and enter the PSM where they participate in formation of gene expression stripes with sharp boundaries, as described above. Notably, for some intermediate region of the PSM, cell mixing would be still fast enough to affect synchronization while the wavelength of gene expression pattern is shortening. Because coupling between oscillators influences the wavelength of gene expression stripes (Ares et al., 2012; Jörg et al., 2015; Murray et al., 2011) and cell mixing extends the range of coupling (Fujiwara et al., 2011; Peruani et al., 2010; Uriu et al., 2013), cell mixing may therefore influence the wavelength of gene expression patterns in this intermediate PSM region. An extended theory that describes the entire PSM and incorporates cell mixing data along the axis will reveal to what extent cell mixing affects the wavelength. Direct experimental tests of these predictions will require means of locally controlling the mixing of cells in the tissue.

Our current analysis suggests that cell mixing in the tailbud is fast enough to influence the dynamics of coupled genetic oscillators in the segmentation clock. A key experiment for testing the theory in living embryos would be to inhibit cell movement with drugs or mutants. A previous study on axial elongation used a drug called blebbistatin to inhibit myosin and block cell movement (Bénazéraf et al., 2010). Using the framework we developed in this paper, one could ask whether impaired cell movement in experimentally treated embryos is enough to slow synchronization dynamics. Previous estimates of the synchronization state (phase order parameter) in the embryo have relied on morphological proxies such as the correct formation of segment boundaries (Riedel-Kruse et al., 2007), which can be modeled by first passage time (Fig. 4C). However, the value of the synchronization state that determines the formation of a normal or defective segment boundary remains unclear. Recently developed live reporters for oscillatory proteins (Delaune et al., 2012; Soroldoni et al., 2014), which should allow direct measurement of the synchronization state and dynamics, are therefore key to testing the theory.

In summary, our study provides a rigorous and systematic framework to investigate cell mixing in one embryological context in which the timescale of cell mixing can be faster than that of intercellular signaling. Relative cell movement may also influence intercellular signaling in other contexts (Uriu et al., 2014), for example in collective migration or gastrulation, or in cultured cell populations with Delta-Notch signaling (Matsuda et al., 2015; Tsiairis and Aulehla, 2016). In addition, for cells under signaling gradients, the relative timescales between mixing and cell type specification by signaling would be important for patterning (Xiong et al., 2013). The ratio of timescales between mixing and signaling determines the impact of mixing (Uriu et al., 2013). In general, quantification of the mixing timescale from imaging data will be simpler than the signaling timescale. Approaches to quantify the influence of cell movement on signaling such as those presented here will be important to understand other similar processes in development and disease.

## MATERIALS AND METHODS

### Imaging setup

Time-lapse imaging data were from Bhavna et al. (2016).

### Cell-tracking algorithm and validation

A gradient vector diffusion algorithm (Li et al., 2007) was used for detecting positions of cell nuclei. Parameter values are listed in Table S4. For cell tracking, the algorithm proposed in Sbalzarini and Koumoutsakos (2005) was used (Supporting Text). Validation of these two algorithms was performed according to Bhavna et al. (2016), using synthetic images and three images of chimeric embryos (Supporting Text). In addition, a recently proposed nuclear segmentation algorithm (Bhavna et al., 2016) was applied to the imaging data to test whether it gave similar MSDD time series (Fig. S5).

### Cell density measurement

The number of cell nuclei in a three dimensional box (42×42×20 μm^3^) (Fig. S7) was counted and divided by the volume of the box. The box was located 20 μm away from epithelial tissues to fill the entire region of the box with mesenchymal cells.

### Velocity vector in laboratory reference frame

Velocity vectors for calculation of directional derivative and strain rate tensor were defined as(7)
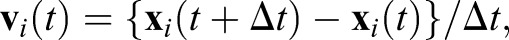
where **x***_i_*(*t*) is the position of cell *i* at time *t* obtained by the tracking algorithm. Δ*t* was set=5 (min) to avoid seeing only the fluctuation of a cell nucleus. The same definition of velocity was used in simulations of cell movement.

### Voronoi tessellation

A three dimensional Voronoi tessellation algorithm in MATLAB R2014b ‘delaunayn’ was applied to nuclear position data to determine neighbor relations among cells. Distances between Voronoi neighbors were calculated by a MATLAB custom code.

### Measurement of nucleus size

The long axis of a nucleus was visually determined in a *x*-*y* plane of image stacks. For this, each *x*-*y* plane containing the nucleus was visually scanned in *z* direction. When the size of the nucleus reached maximum, the length of its long axis was measured in that plane with the line tool from Fiji.

### Fitting by ABC MCMC

The algorithm proposed in Marjoram et al. (2003) was used. Parameter values are listed in Table S5 (see also Supporting Text). The custom code for ABC MCMC was written in C language.

### Strain rate tensor

To construct a continuum velocity vector field **v**(*t*, **x**) in a three-dimensional space from the data for cell velocity vectors **v***_i_*(*t*, **x***_i_*), the smoothed particle hydrodynamics (SPH) approach was used. Strain rate tensor was then computed using the continuum velocity vector field (Supporting Text).

### Mean squared difference of displacement vectors

Boxes of size 48×48×*z* μm^3^ (*z*=47 for 15ss, 61 for 16ss and 42 for two 17ss embryos) were set in PSM and tailbud (Fig. 2A), and cells within each box during imaging period were used for computation of MSDD using Eqn 3.

### Numerical integration of differential equations

The stochastic differential Eqns 4 and 5 were solved with the Euler-Maruyama method with time step *Δt*=0.01. The custom code was written in C language.
